# Assessing Aβ‐independent effects of Module 42 on immune function in vitro

**DOI:** 10.1002/alz.71215

**Published:** 2026-02-25

**Authors:** Ishita Ajith, Souvika Bakshi, Emma Mead, Opher Gileadi, Vittorio L. Katis, Paul E. Brennan, Emory‐Sage‐SGC‐Jax TREAT‐AD Center, Katerina O. Gospodinova

**Affiliations:** ^1^ Centre for Medicines Discovery Oxford UK; ^2^ Institute for Stroke and Dementia Research (ISD) University Hospital LMU Munich Munich Germany; ^3^ Alzheimer's Research UK Oxford Drug Discovery Institute Oxford UK; ^4^ Centre for Molecular Medicine Karolinska Institutet and Karolinska Hospital Solna Stockholm Sweden; ^5^ Department of Medicine Solna Structural Genomics Consortium Karolinska Institutet Stockholm Sweden; ^6^ adknowledgeportal.org/TREATAD‐ESS‐Center

**Keywords:** Alzheimer's disease, immune function, M42 proteins, matrisome

## Abstract

**INTRODUCTION:**

A deep multi‐omic analysis of *post mortem* human brains has identified a new co‐expression protein network – Module 42 (M42), strongly corelated with Alzheimer's disease (AD) pathology. M42 comprises 32 transmembrane and extracellular matrix (ECM)‐associated proteins, including the amyloid precursor protein (APP) and apolipoprotein E (apoE), and its members have been implicated in amyloid beta (Aβ) pathology. We systematically evaluated the Aβ‐independent effects of M42 on immune function in vitro.

**METHODS:**

Recombinant M42 proteins were expressed and purified. Their effects on phagocytosis, intracellular signaling, and cell viability were assessed in human induced pluripotent stem cell‐derived macrophages.

**RESULTS:**

Treatment with Midkine (MDK) reduced phagocytosis, while treatment with the ectodomain of Transmembrane protein with EGF‐like and two follistatin‐like domains 2 (TMEFF2) had the opposite effect. Both proteins promoted intracellular Ca^2+^ signaling, and TMEFF2 also suppressed Syk kinase activity. No M42 proteins had an effect on viability.

**DISCUSSION:**

Our results suggest an additional role for M42 in AD via regulating immune functions.

**Highlights:**

- We tested M42 proteins for their effects on immune functions in vitro.
- Five proteins altered phagocytosis, and seven altered Ca^2+^ signaling.
- MDK and TMEFF2 ectodomain had an effect on both phagocytosis and Ca^2+^ signaling.

## 1 BACKGROUND

Dementia poses a global health challenge, with over 55 million people currently living with dementia worldwide. According to the World Health Organization, these numbers are predicted to rise to up to 78 million by 2030. Alzheimer's disease (AD), the most common cause of dementia, is a progressive neurodegenerative disorder clinically characterized by cognitive decline, memory loss, and a range of neuropsychiatric symptoms including depression, anxiety, sleep disturbances, social isolation, hallucinations, and delusions. The deposition of extracellular amyloid beta (Aβ) plaques and neurofibrillary hyperphosphorylated tau tangles in the brain is thought to drive AD initiation and progression, respectively.^1^ Recently approved anti‐amyloid immunotherapies, such as lecanemab and donanemab, have shown promise in slowing the rate of cognitive decline while clearing Aβ from the brain effectively.^2^ Although these therapies provide evidence for the central role of Aβ in AD pathogenesis, the modest clinical benefits and adverse events observed (e.g., amyloid‐related imaging abnormalities or ARIA) highlight the limitations of targeting amyloid alone.

Large‐scale multi‐omic approaches have uncovered diverse molecular mechanisms beyond Aβ and tau contributing to AD pathology, including neuroinflammation, altered proteostasis, and neurovascular dysfunction.^3^, ^4^ In 2022, the Accelerated Medicines Partnership for Alzheimer's Disease (AMP‐AD) consortium published a deep multilayer analysis of over 1000 *post mortem* human brain samples from individuals diagnosed with AD, asymptomatic AD (AsymAD), and control subjects.^4^ Through integrating genomic, transcriptomic, and proteomic approaches, this study identified novel protein network modules strongly correlating with AD pathology and cognition. Among these, Module 42 (M42) – the “matrisome” – showed the strongest correlation with pathology. M42 protein abundance, but not RNA levels, was found to be highly elevated in *post mortem* brain samples from AsymAD and AD cases and strongly influenced by the *APOE* genotype, suggesting a role in early disease processes. Increased levels of several members of the module have also been detected in cerebrospinal fluid (CSF) samples from AD patients and proposed as novel candidate biomarkers.^5^, ^6^ For instance, SPARC‐related modular calcium‐binding protein 1 (SMOC1) CSF levels were found to reliably classify AD cases versus non‐AD controls, as well as to predict Aβ/tau levels across multiple cohorts.^6^ Additionally, M42 was found to be preserved in mouse models of AD, including the 5xFAD^3^ and CRND8^7^ mice, exhibiting elevated levels with age and compared to control animals.

**RESEARCH IN CONTEXT**

1. **Systematic review**: We searched PubMed and found no reports systematically evaluating the effects of M42 proteins on cellular responses in vitro, including immune function in particular. However, previous reports addressed the role of individual module members in neuroinflammation as well as in AD.
2. **Interpretation**: In agreement with previous reports in the literature, our findings suggest a role for M42 in modulating immune responses. We highlight a novel role for MDK and TMEFF2 in regulating phagocytosis and intracellular signaling.
3. **Future directions**: We have generated tools that can enable profiling of M42 activity across different cell types. We propose that evaluating the effects of the module as a whole, particularly in complex in vitro systems, may be especially informative given its non‐cell‐autonomous effects.

M42 comprises a network of predominantly secreted and transmembrane extracellular matrix (ECM)‐associated proteins, including the amyloid precursor protein (APP) and apolipoprotein E (apoE). Independent studies have demonstrated that several M42 members are enriched in Aβ plaques and colocalize with cerebrovascular amyloid and tau neurofibrillary tangles in *post mortem* brain samples and AD mouse models.^3^, ^7^, ^8^ Furthermore, some module members, such as Secreted frizzled‐related protein 1 (SFRP1), Midkine (MDK), Pleiotrophin (PTN), and Netrin‐1 (NTN1), physically interact with Aβ, further promoting its aggregation both in vitro and in vivo.^3^, ^5^, ^7^ The direct effect on Aβ aggregation has been attributed to the presence of heparan sulfate and glycosaminoglycan‐binding domains, potentially mediating the interaction between M42 proteins and Aβ. In addition to directly affecting Aβ aggregation, other module members (e.g., Transmembrane protein with EGF‐like and two follistatin‐like domains 2 (TMEFF2), NTN1, and Spondin‐1 (SPON1)) have been shown to influence plaque deposition through modulating APP processing and/or cellular responses.^9^, ^10^, ^11^

Taken together, these data indicate a strong relationship between M42 and AD pathogenesis potentially through influencing Aβ plaque deposition. However, the role of M42 on modulating downstream cellular responses remains poorly explored. In this study, we examined the Aβ‐independent effects of M42 proteins on immune function using human induced pluripotent stem cell (hiPSC)‐derived macrophages as an in vitro model. Following a systematic analysis of individual module members, we identified High‐temperature requirement A1 (HTRA1), Dystroglycan (DAG1), TMEFF2, MDK, and Glypican‐5 (GPC5) as phagocytosis modulators in vitro. Subsequently, we showed that these effects were cargo‐dependent and, in the case of MDK and TMEFF2, likely mediated by altered intracellular Ca^2+^ signaling. Importantly, the observed effects on microglial function were not the result of altered viability. This work highlights a role for M42 in contributing to AD pathology through modulating immune function independent of Aβ deposition.

## 2 METHODS

### 2.1 Protein expression and purification

Expression vectors containing DNA encoding full‐length and/or specific domains were generated for each M42 protein. His and Avi tags were introduced at the N‐ and/or C‐terminal to enable protein purification. Detailed plasmid maps can be found in the corresponding Target Enablement Package (TEP) listed in Supplementary Table S1 for each protein. All plasmids have been deposited in Addgene (https://www.addgene.org/depositor‐collections/treat‐ad/) and are available upon request.

Recombinant proteins were produced in *S. frugiperda* Sf9 insect cells via baculovirus infection or Expi293F cells via transient transfection in an endotoxin‐free manner, following published TEP protocols. Produced regions, expression system used and links to TEP protocols for each protein are summarized in Supplementary Table S1. In short, Sf9 cells were grown in Sf900‐II SFM Medium (10902096, Thermo Fisher Scientific) at 27°C. When cells reached a density of 2 × 10^6^ cells/mL, the medium was supplemented with 2% FBS (v/v) and cells infected with 1 in 200 volume of P2 baculovirus stock. Cell supernatants were harvested 3 days after infection for protein purification. Alternatively, Expi293F were grown in FreeStyle 293 Expression Medium (12338018, Thermo Fisher Scientific) and transiently transfected upon reaching a cell density of 1.8–2 × 10^6^ cells/mL with a linear polyethylenimine/plasmid (6:1) mixture. Medium was supplemented with sodium butyrate (12 mM) to enhance protein production. Cell supernatants were collected 5 days after transfection for protein purification.

Following collection, cell culture supernatants were incubated with pre‐equilibrated TALON (Sf9‐based expression) or Ni‐sepharose (Expi293F‐based expression) beads and purified using immobilized metal‐affinity chromatography (IMAC). Heparin‐binding proteins (MDK, PTN, SFRP1, and Frizzled related protein (FRZB)) were further purified using heparin affinity chromatography (HiTrap Heparin HP) and the rest via size exclusion chromatography using a HiLoad Superdex S200 column. Additional ion exchange chromatography (Source Q) was performed when necessary. Protein purity was assessed by SDS‐PAGE, identity confirmed by intact mass spectrometry, and concentration determined by ultraviolet spectroscopy. Purified proteins were aliquoted, flash‐frozen, and stored at −70°C. Detailed purification protocols for each protein are provided in the corresponding TEPs listed in Supplementary Table S1.

Proteins that could not be expressed in sufficient quantities and/or good enough purity (Supplementary Table S2) were excluded from subsequent functional analyses in vitro, and commercially available full‐length proteins were purchased instead where possible. These include HTRA1 (2916‐SE‐020, Novus Biologicals; *E. coli* expression system), Fms‐related tyrosine kinase 1 (FLT1; FCL1058B, G&P Biosciences; mammalian expression system), SPON1 (3135‐SP‐025/CF, R&D Systems; mammalian expression system), GPC5 (2607‐G5‐050/CF, R&D Systems; mammalian expression system), and Olfactomedin‐like 3 (OLFML3; Acro Biosystems, OL3‐H52H4‐100ug; mammalian expression system). APP and apoE were also excluded from any downstream functional analyses due to being widely studied by the scientific community.

### 2.2 Endotoxin levels

Endotoxin levels of in‐house purified recombinant M42 proteins (Table S1) were assessed using the Pierce Chromogenic Endotoxin Quant Kit (A39552, Thermo Fisher Scientific) according to the manufacturer's instructions. In brief, all reagents were equilibrated to room temperature (RT) prior to use, and proteins were diluted to 1 µg/mL in endotoxin free water. Standards were diluted as per instructions. Blanks, diluted standards, and proteins (50 µL) were dispensed in a 96‐well plate in triplicate (standards) or duplicate (blanks and protein samples). Lyophilized Amebocyte Lysate (LAL) was prepared according to the manufacturer's instructions and 50 µL added to each well. Samples were mixed by gently swirling the plate, and the reaction was incubated at RT for 25 min. In the meantime, the chromogenic substrate, provided with the kit, was reconstituted and heated to 37°C for 5 to 10 min. Prewarmed substrate (100 µL) was added to the each well, mixed gently, and incubated for a further 6 min. The reaction was then stopped by adding 50 µL of 25% acetic acid. Absorbance at 450 nm was measured immediately using the PHERAstar FSX (BMG Labtech). Endotoxin levels for all recombinant proteins were below the threshold considered acceptable for cell culture (<1 EU/mL), with all proteins except PTN measuring below 0.1 EU/mL – the level of endotoxin recommended for immune cell‐based in vitro assays. Determined endotoxin levels are shown in Supplementary Table S3.

### 2.3 Culture of hiPSC‐derived macrophages

The hiPSC line BIONi010‐C (BioSample ID: SAMEA3158050, ECACC ID: 66540023) was obtained from Bioneer. Human iPSCs were maintained on human embryonic stem cell‐qualified Geltrex‐coated plates (A1569601, Gibco) in complete mTeSR™1 medium (85850, STEMCELL Technologies). Upon reaching 80% confluency, hiPSCs were passaged as clumps using 0.5mM Ultrapure EDTA (15575020, Life Technologies) in DPBS (14190250, Gibco). Human iPSCs were passaged at least twice and maintained for at least a week before being differentiated. Embryoid bodies (EBs) were generated by seeding 4 × 10^6^ hiPSCs per well in AggreWell‐800 plates (34811, STEMCELL Technologies) and maintained in mTeSR™1 medium supplemented with (Bone morphogenetic protein‐4; 50 ng/mL, PHC9534, Life Technologies), (Vascular endothelial growth factor; 50 ng/mL, PHC9391, Life Technologies), and (Stem cell factor; 20 ng/mL, 130‐096‐695, Miltenyi Biotec) for 3 days. EBs were subsequently transferred to low‐attachment plates (657970, Greiner Bio‐One) and maintained for another 3 days. EBs were then transferred to 0.1% gelatin type B (G1393‐100ML, Sigma) coated T175 flasks containing X‐VIVO15 medium (LZBE02–060F, Lonza) supplemented with GlutaMAX (2 mM, 35050038, Life Technologies), Macrophage colony‐stimulating factor (M‐CSF; 100 ng/mL, PHC9501, Life Technologies), Interleukin‐3 (IL‐3; 25 ng/mL, PHC0031, Life Technologies), 2‐mercaptoethanol (50 µM, 31350010, Gibco), and penicillin/streptomycin (100 U/mL and 100 µg/mL, 15140122, Sigma). Macrophage precursors were harvested weekly and differentiated to macrophages for a minimum of 7 days in X‐VIVO 15 medium containing M‐CSF (100 ng/mL), GlutaMAX (2 mM), and penicillin/streptomycin (100 U/mL each). Half‐medium change was performed 3 days after plating.

### 2.4 Phagocytosis assay

SH‐SY5Y cells (CRL‐2266, ATCC) were cultured in DMEM/F12 medium (11320074, Gibco) supplemented with 10% FBS (F4135, Sigma‐Aldrich). Cells were harvested using TrypLE Express (12604013, Gibco), collected in DPBS, and centrifuged at 400 × g for 5 min. Cell pellets were resuspended in Live Cell Imaging Solution (LCIS, A14291DJ, Invitrogen) and fixed in 2% paraformaldehyde for 10 min at RT. Fixed cells were then washed with DPBS and centrifuged at 1200 × g for 7 min. Cell pellets were resuspended in LCIS and transferred to low protein‐binding tubes prior to labeling with pHrodo iFL Red STP Ester (P36011, Life Technologies, 12.5 µg per million cells) for 30 min at RT. Labeled apoptotic SH‐SY5Y cells were spun and washed twice with DPBS by centrifuging at 1200 × g for 5 min. Pellets were stored at −70°C for up to 30 days.

Macrophage precursors were plated at a density of 3 × 10^4^ cells/well in 96‐well PhenoPlates (Revvity) and differentiated for 8 days. For phagocytosis assays, all treatments were carried out in phenol red‐free X‐VIVO 15 (04–744Q, Lonza) supplemented with Glutamax (2 mM), penicillin (100U/mL), and streptomycin (100 µg/mL) (subsequently referred to as “phagocytosis medium”). pHrodo‐labeled SH‐SY5Y cells were thawed, resuspended to 1.2 × 10^6^ cells/mL in phagocytosis medium. M42 proteins were diluted in phagocytosis medium to 6 µg/mL, 2 µg/mL, or 200 ng/mL (2× final concentrations). Macrophage medium was replaced with 50 µL of the corresponding protein or vehicle control treatment followed by addition of 50 µL pHrodo‐labeled SH‐SY5Y cells (6 × 10^4^ cells/well). For measuring zymosan phagocytosis, 50 µL pHrodo‐labeled zymosan Bioparticles (20 µg/mL; P35364, Thermo Fisher Scientific) were added to treated wells and corresponding controls. Pretreatment with cytochalasin D (1.5 µM, 11330‐1 mg‐CAY, Cayman) for 1 h at 37°C prior to the addition of labeled apoptotic bodies or zymosan particles served as a negative control. All treatments were run in triplicate.

Phagocytosis was monitored using the Incucyte SX5. Images were captured hourly for 24 h (three images/well, 10 × objective, 300 ms acquisition) using phase and orange channels (546 to 568 nm excitation, 576 to 639 nm emission). The first time point was acquired following 30 min equilibration. Data were exported as macrophage confluency (phase) and total integrated intensity (TII, pHrodo fluorescence). Phagocytic capacity was calculated as TII/confluency and quantified as area under the curve (AUC) using GraphPad Prism.

### 2.5 Ca^2+^ Signaling assay

Macrophage precursors were plated at a density of 1 × 10^4^ cells/well in optically clear bottom CellCarrier 384‐well plates (Perkin Elmer) and differentiated for 7 days.

For measuring intracellular Ca^2+^ levels, all treatments were carried out in 1x Hanks' Balanced Salt Solution buffer (HBSS; H8264, Sigma) supplemented with 20 mM HEPES (HBSS/H). Macrophage medium was removed and replaced with 25 µL of 4 µM Calcium‐6 dye (R8190, Molecular devices) prepared in the HBSS/H buffer. Macrophages were incubated for 1 h at 37°C, 5% CO_2_. Protein treatments were prepared in HBSS/H buffer at 5× their final concentration (e.g., 15 µg/mL for 3 µg/mL final concentration) in 384‐well plates (“source plates”). Histamine (6 µM final concentration) was used as a positive control.

Calcium responses were measured using a FLIPR Tetra system (Molecular Devices). Baseline fluorescence was recorded for 1 min using 470‐ to 495‐nm excitation and 515‐ to 575‐nm emission. Treatments were added to the Calcium‐6‐labeled macrophages. Responses were recorded for 5 min at 1‐s intervals. Data were analyzed using ScreenWorks software and expressed as AUC, normalized to baseline (mean relative fluorescence units [RFU] recorded in the first 1 min). All conditions were run in quadruplicates.

### 2.6 Phospho‐Spleen tyrosine kinase (SYK) homogeneous time‐resolved fluorescence (HTRF) assay

Macrophage precursors were seeded at 4 × 10^4^ cells/well in 96‐well plates and differentiated for 8 days. Phospho‐SYK (Tyr525/526) and total SYK levels were measured using HTRF kits (total SYK: 64SYKTPEG, phospho‐SYK Y525/526: 64SYKY525PEG, PerkinElmer) following the manufacturer's instructions. Macrophage medium was replaced with medium containing 3 µg/mL MDK or TMEFF2 and cells were incubated for 1 h at 37°C, 5% CO_2_. For stimulation experiments, macrophages were further stimulated with Triggering receptor expressed on myeloid cells 2 (TREM2)‐activating antibody (5 µg/mL, AF1828, R&D Systems) or IgG control (5 µg/mL, AB‐108‐C, R&D Systems) for 5 min at 37°C, 5% CO_2_. Following treatments, cells were lysed in kit‐provided lysis buffer supplemented with cOmplete protease inhibitor cocktail (4693132001, Merck) and PhosSTOP (4906845001, Merck) for 30 min at RT with orbital shaking (220 rpm). Cell lysates were transferred to ProxiPlate‐384 Plus plates (6008260, PerkinElmer) and incubated overnight with premixed Eu^3^^+^‐cryptate and d2 antibodies diluted in detection buffer provided in the kit. Lysis buffer‐only controls were included on each plate. HTRF signals were measured using a PHERAstar FSX (BMG Labtech) with the HTRF optic module, detecting emissions at 665 and 620 nm. Data were exported as RFU for each wavelength, and signal‐to‐noise ratios were calculated in GraphPad Prism. All treatments were performed in triplicate.

### 2.7 CellTiter‐Glo viability assay

Macrophage precursors were seeded at 4 × 10^4^ cells/well in opaque walled 96‐well plates (15042, Thermo Fisher Scientific) and differentiated for 8 days.

M42 proteins were diluted in macrophage medium to 3 µg/mL, 1 µg/mL, or 100 ng/mL. Macrophage medium was replaced with corresponding treatments, and cells were incubated for 24 h at 37°C, 5% CO_2_. Cell viability was assessed using the CellTiter‐Glo Luminescent Cell Viability Assay (G7570, Promega) according to the manufacturer's instructions. Briefly, plates were equilibrated to RT for 30 min before adding an equal volume of CellTiter‐Glo reagent to each well. Plates were mixed on an orbital shaker for 2 min to induce cell lysis, then incubated at RT for 10 min to stabilize the luminescent signal.

Luminescence was measured using a PHERAstar FSX plate reader (BMG Labtech). Data were exported as relative luminescence units (RLU) and analyzed using GraphPad Prism. All conditions were performed in triplicate.

### 2.8 Statistical analysis

All data are presented as mean ± standard deviation (SD) from independent biological experiments. Statistical analyses were performed using GraphPad Prism 10. Comparisons between groups were assessed using paired *t*‐test (two groups) or repeated measures one‐way ANOVA followed by Dunnett's multiple comparisons test (one independent variable; multiple groups). Specific statistical tests and post hoc analyses used are indicated at the end of each figure legend. *P* values are reported in the text where relevant. Statistical significance was set at *p* < 0.05.

## 3 RESULTS

M42 proteins have been previously implicated in Aβ plaque formation, driving aggregation in vitro and plaque accumulation in vivo.^7^ Given the established role of neuroinflammation in AD pathogenesis,^12^ we investigated the effects of M42 protein members on immune function independent of Aβ.

HiPSCs derived from a healthy donor were differentiated to tissue‐resident macrophages using a well‐established protocol.^13^ Endotoxin‐free full‐length or extracellular domains of M42 proteins were expressed and purified in house or purchased. Individual recombinant proteins were tested at three concentrations – 3 µg/mL, 1 µg/mL, and 100 ng/mL – to assess their effects on key immune functions and intracellular signaling in hiPSC‐derived macrophages.

### 3.1 M42 members alter phagocytosis of apoptotic neurons in vitro

**FIGURE 1:**
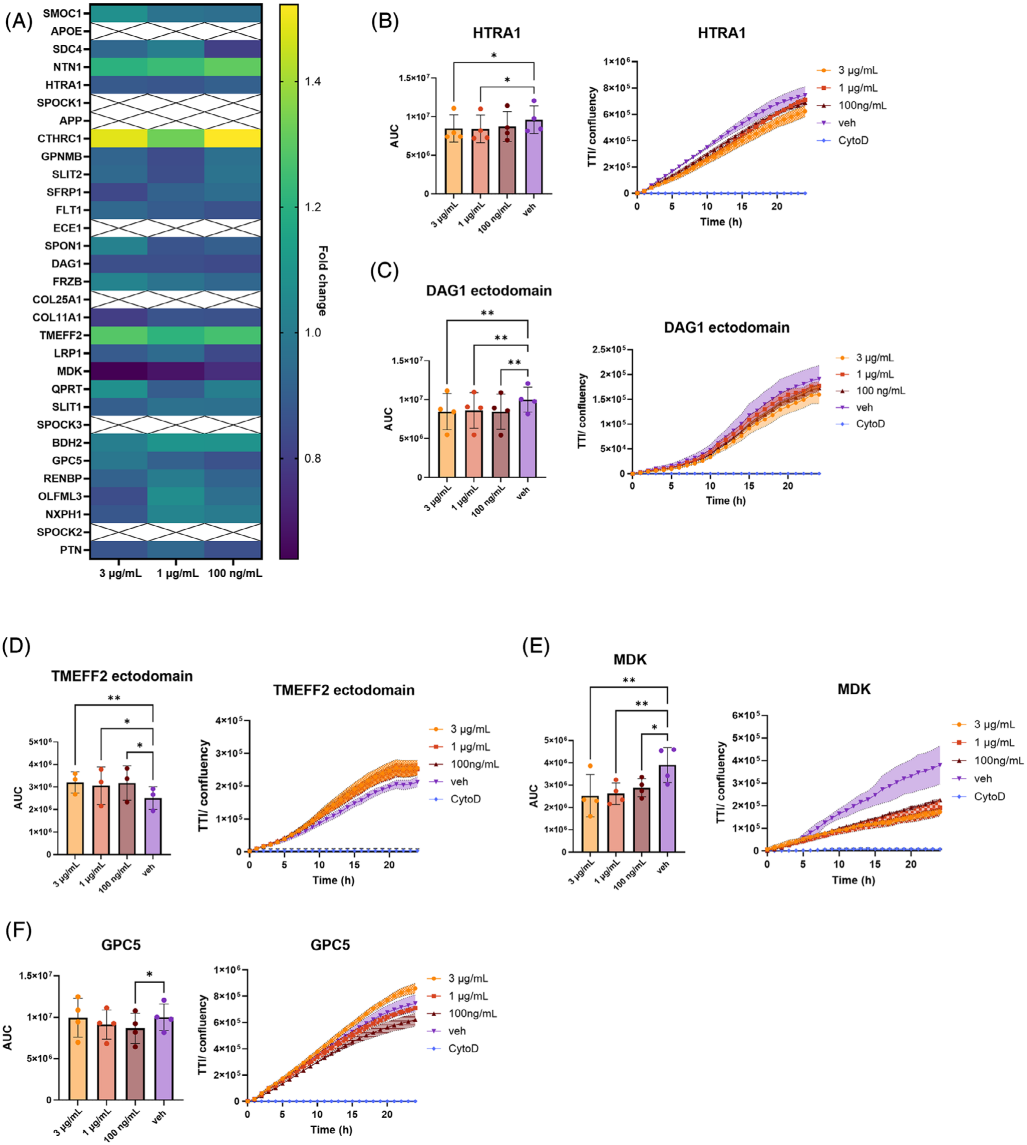
Assessing the effect of M42 recombinant proteins on phagocytosis of apoptotic SH‐SY5Y cells in human induced pluripotent stem cell (hiPSC)‐derived macrophages. (A) Summary graph showing fold change in total phagocytosis assessed over 24 h relative to control (vehicle‐treated cells) across all tested proteins at three concentrations: 3 µg/mL, 1 µg/mL, and 100 ng/mL. (B–F) Phagocytosis following treatment with HTRA1 (B), DAG1 ectodomain (C), TMEFF2 ectodomain (D), MDK (E), and GPC5 (F). Left panels show comparison between phagocytosis levels in vehicle‐ and protein‐treated hiPSC macrophages quantified by area under the curve (AUC). Right panels show a representative trace from one biological repeat (*n* = 3 technical replicates). Levels of phagocytosis are measured as total integrated intensity of pHrodo‐labeled apoptotic neurons normalized to the confluency of macrophage layer (TII/confluency). Cytochalasin D (CytoD, 10 µM) treatment was included in every experiment as a negative control. *N* = 3 to 4 independent biological experiments (one or two independent macrophage factory set‐ups; three technical replicates/biological repeat). Error bars correspond to mean ± SD. Repeated measures one‐way ANOVA followed by Dunnett's multiple comparisons test. *p < 0.05; **p < 0.01.

First, we evaluated the effect of treatment with individual recombinant proteins on phagocytosis as a key immune function implicated in brain homeostasis. HiPSC macrophages were treated with recombinant M42 proteins at the indicated concentrations – 3 µg/mL, 1 µg/mL, and 100 ng/mL, in the presence of pHrodo‐labeled apoptotic SH‐SY5Y neurons. Uptake was monitored over 24 h using live‐cell high‐content imaging. Total levels of phagocytosis were quantified and data summarized as fold changes relative to vehicle‐treated controls (Figure 1A). Significant effects on phagocytosis were observed following treatment with five M42 proteins – full‐length HTRA1 (Figure 1B, *F* = 6.217, *p* = 0.0142), DAG1 ectodomain (Figure 1C, *F* = 12.83, *p* = 0.0013), TMEFF2 ectodomain (Figure 1D, *F* = 9.515, *p* = 0.0107), full‐length MDK (Figure 1E, *F* = 9.605, *p* = 0.0036), and full‐length GPC5 (Figure 1F, *F* = 5.982, *p* = 0.0158). Treatment with HTRA1, DAG1 ectodomain, MDK, and GPC5 decreased phagocytosis levels. MDK showed the most prominent dose‐dependent effect, reducing overall uptake of apoptotic neurons by 50% at 3 µg/mL. Conversely, treatment with TMEFF2 ectodomain led to a significant increase in phagocytosis – by up to 25% on average when tested at 3 µg/mL. Full‐length GPC5 only had a significant impact on phagocytosis at 100 ng/mL. Although treatment with Collagen triple helix repeat‐containing protein 1 (CTHRC1) increased phagocytosis by up to 50% on average when tested at 3 µg/mL and 100 ng/mL, these effects were variable and did not reach statistical significance (Supplementary Figure S1, *F* = 3.946, *p* = 0.0719).

The uptake of apoptotic neuronal bodies relies on the exposure of phosphatidylserine and was previously shown to be largely driven by TREM2 signaling in our hiPSC‐derived macrophage model.^14^ We thus sought to investigate whether the observed effects with these five M42 proteins on phagocytosis were cargo‐dependent. Using a similar experimental set‐up, we profiled the effect of treatment with 3 µg/mL of recombinant M42 proteins on the uptake of pHrodo‐labeled zymosan particles in hiPSC‐derived macrophages. Total levels of phagocytosis following treatment are expressed as fold change relative to corresponding vehicle‐treated cells and summarized in Supplementary Figure S2A. Unlike the effects we observed on the uptake of apoptotic bodies, treatment with full‐length HTRA1, MDK, and GPC5, and the ectodomains of TMEFF2 and DAG1 had no significant impact on the phagocytosis of zymosan particles (Supplementary Figure S2B–F). In fact, none of the M42 protein members tested had a significant impact in this phagocytosis paradigm. Taken together, these results suggest that M42 proteins secreted in the extracellular space selectively modulate the phagocytosis of apoptotic bodies, potentially downstream of signaling receptors such as TREM2, rather than exerting broader effects on phagocytic activity.

### 3.2 M42 members modulate intracellular signaling in hiPSC‐derived macrophages

**FIGURE 2:**
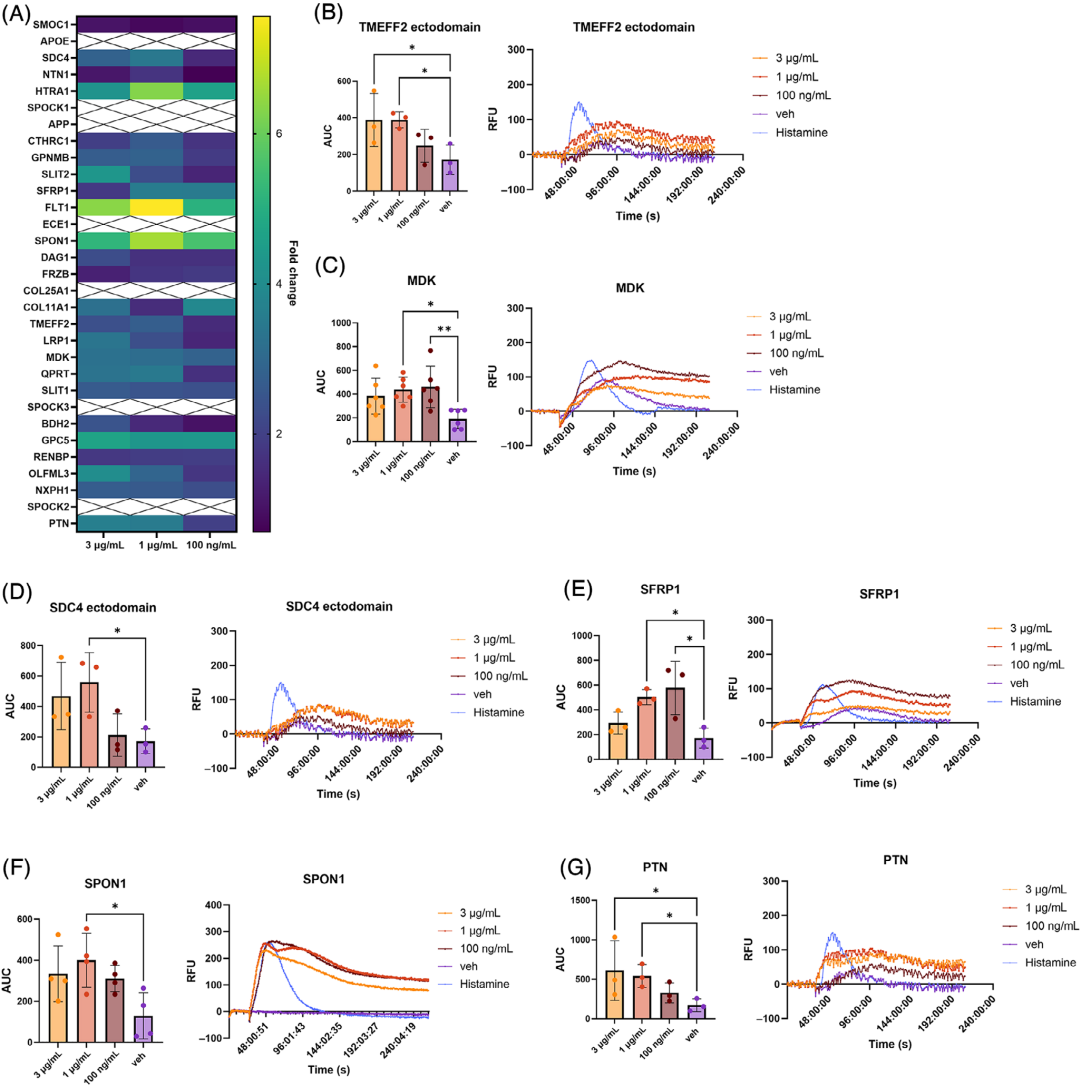
Assessing the effect of M42 recombinant proteins on intracellular Ca^2+^ signaling in human induced pluripotent stem cell‐derived macrophages. (A) Summary graph showing the fold change in total Ca^2+^ levels following the addition of recombinant M42 proteins. (B–G) Intracellular Ca^2+^ levels following treatment with TMEFF2 ectodomain (B), MDK (C), SDC4 ectodomain (D), SFRP1 (E), SPON1 (F), and PTN (G). Left panels represent intracellular Ca^2+^ levels defined as area under the curve (AUC). Right panels show representative traces from one biological repeat (n = 3 technical replicates). Intracellular Ca^2+^ levels are measured as relative fluorescent units. Histamine (6 µM) was used as a positive control. *N* = 3 to 6 independent biological experiments (two independent macrophage factory set‐ups; four technical replicates/biological repeat). Error bars correspond to mean ± SD. Repeated measures one‐way ANOVA followed by Dunnett's multiple comparisons test. *p < 0.05, **p < 0.01.

Gene ontology analysis previously implicated M42 members in calcium ion binding.^4^ To explore whether these proteins influenced Ca^2+^ signaling, we examined changes in intracellular Ca^2+^ levels in hiPSC macrophages following treatment with recombinant M42 proteins (Figure 2). Cells were pre‐incubated with Ca^2+^ sensing fluorescent dye, and intracellular Ca^2+^ currents were monitored over time following the addition of individual proteins. The obtained results are expressed as fold changes relative to corresponding vehicle‐treated controls (Figure 2A). Among the five proteins that had a significant impact on the levels of phagocytosis of apoptotic neurons, TMEFF2 ectodomain and MDK also evoked a significant Ca^2+^ response (Figure 2B,C: TMEFF2, *F* = 4.859, *p* = 0.0479; MDK, *F* = 5.191, *p* = 0.0117). TMEFF2 ectodomain exerted a dose‐dependent effect with 3 and 1 µg/mL producing a significant response (Figure 2B). In contrast, MDK showed the strongest response at the lowest concentration tested – 100 ng/mL (Figure 2C). Treatment with HTRA1 was associated with a marginally significant increase in intracellular Ca^2+^ levels (Supplementary Figure S3A, *F* = 3.735, *p* = 0.0541), while the addition of DAG1 ectodomain and full‐length GPC5 had no significant effects (Supplementary Figure S3B,C: DAG1, *F* = 1.170, *p* = 0.3963; GPC5, *F* = 1.511, *p* = 0.2770).

In addition to these, we identified four other M42 proteins that evoked a significant increase in Ca^2+^ response: the ectodomain of Syndecan‐4 (SDC4; Figure 2D, *F* = 4.844, *p* = 0.0482), SFRP1 (Figure 2E, *F* = 7.468, *p* = 0.0189), SPON1 (Figure 2F, *F* = 4.509, *p* = 0.0342), and PTN (Figure 2G, *F* = 5.845, *p* = 0.0326). Full‐length PTN increased intracellular Ca^2+^ levels in a dose‐dependent manner (Figure 2G), while the ectodomain of SDC4 and full‐length SPON1 had the most pronounced effects at 1 µg/mL (Figure 2D,F). Meanwhile, SFRP1, like MDK, showed an inverse relationship, with 100 ng/mL evoking the strongest Ca^2+^ response (Figure 2E). We also observed a significant effect following treatment with the olfactomedin (OLF) domain of OLFML3 (Supplementary Figure S4A, *F* = 7.032, *p* = 0.0098). In contrast, treatment with commercially available full‐length OLFML3 did not elicit any significant effect on intracellular Ca^2+^ release (Figure 4B, *F* = 2.216, *p* = 0.1557). Since these proteins differed both in length and expression system (the OLF domain was produced in‐house from Sf9 insect cells, while the full‐length version was obtained commercially and purified from HEK293 cells), this protein was omitted from further evaluation.

**FIGURE 3:**
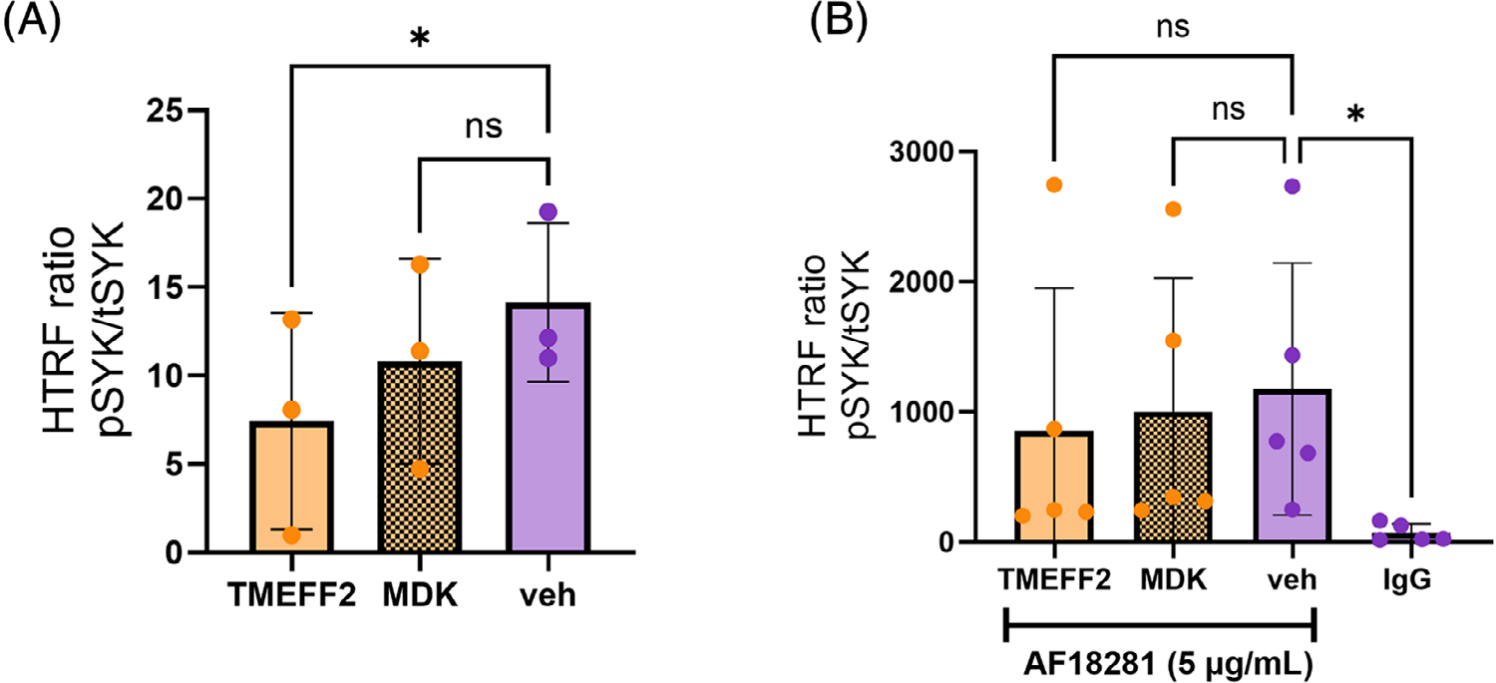
Assessing the effect of TMEFF2 ectodomain and MDK on SYK phosphorylation in human induced pluripotent stem cell (hiPSC)‐derived macrophages. HiPSC macrophages were treated with TMEFF2 ectodomain and MDK (3 µg/mL). Total and phosphorylated SYK levels were evaluated using homogeneous time‐resolved fluorescence both at baseline (A) and following stimulation with a TREM2‐activating antibody – AF18281 (5 µg/mL) (B). IgG isotype control was included as a control for the conditions including stimulation with AF18281. *N* = 3 to 5 independent biological experiments (one independent macrophage factory set‐up; two technical replicates/ biological repeat). Error bars correspond to mean ± SD. Repeated measures one‐way ANOVA followed by Dunnett's multiple comparisons test. *p < 0.05.

SYK phosphorylation and activation plays a crucial role in mediating both Ca^2+^ signaling and phagocytosis in immune cells, downstream of signaling receptors such as TREM2. Given the concordant effects of MDK and TMEFF2 ectodomain on intracellular Ca^2+^ levels and apoptotic neurons uptake, we next assessed their effects on SYK phosphorylation in hiPSC macrophages. Cells were treated with 3 µg/mL of TMEFF2 ectodomain and MDK, both at baseline and prior to stimulation with a TREM2‐activating antibody (Figure 3). At baseline, the ectodomain of TMEFF2 significantly reduced SYK phosphorylation levels compared to those detected in the vehicle control (*p* = 0.0138), while MDK had no significant effect (*p* = 0.1191) (Figure 3A). As previously reported,^14^ TREM2 stimulation with the activating antibody AF18281 (5 µg/mL) led to a significant increase in SYK phosphorylation compared to the IgG isotype control (Figure 3B, *p* = 0.0131). However, pretreatment with TMEFF2 ectodomain or MDK had no significant effects on TREM2‐induced SYK phosphorylation (Figure 3B).

### 3.3 Treatment with M42 has no effect on the viability of hiPSC‐derived macrophages

**FIGURE 4:**
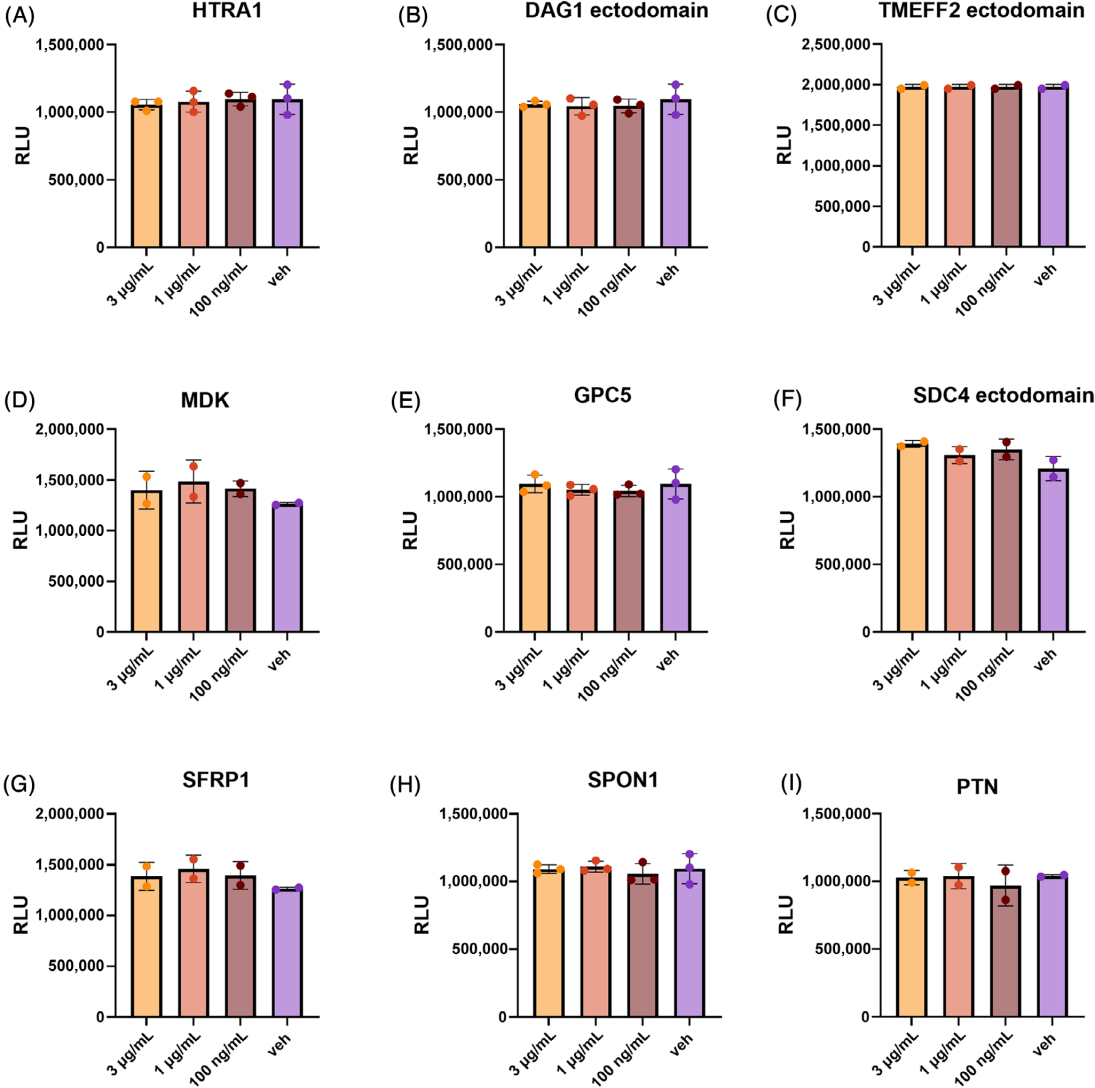
Assessing the effect of M42 protein treatment on viability of hiPSC derived macrophages. Human iPSC macrophages were treated with HTRA1 (A), DAG1 ectodomain (B), TMEFF2 ectodomain (C), MDK (D), GPC5 (E), SDC4 ectodomain (F), SFRP1 (G), SPON1 (H), and PTN (I) at 3 µg/mL, 1 µg/mL, and 100 ng/mL for 24 h. Viability was assessed and expressed as relative luminescence units (RLU). *N* = 2 or 3 independent biological experiments (one independent macrophage factory set‐up; three technical replicates/biological repeat). Error bars correspond to mean ± standard deviation (SD). Repeated measures one‐way ANOVA followed by Dunnett's multiple comparisons test.

Finally, to exclude the possibility that the effects we observed in the functional assays described above were the result of changes in cell health, we assessed the viability of hiPSC macrophages following a 24‐h treatment with full‐length HTRA1, MDK, GPC5, SFRP1, SPON1, PTN, the ectodomains of TMEFF2, DAG1, and SDC4 across the same three concentrations – 3 µg/mL, 1 µg/mL, and 100 ng/mL (Figure 4). None of these proteins had a significant effect on macrophage health at any of the concentrations tested.

## 4 DISCUSSION

Multi‐omic analysis of human *post mortem* brain samples has identified novel co‐expression modules strongly associated with AD.^4^ Among these, M42 comprises a co‐expression network enriched in ECM‐associated proteins, whose protein abundance shows a strong positive correlation with both Aβ accumulation and tau pathology. Hub module proteins include the major AD risk genes *APP* and *APOE*. Notably, M42 protein levels are strongly influenced by the *APOE* genotype, with the ε4 allele associated with a significant increase, particularly in asymptomatic AD *post mortem* brains, suggesting a role in early disease pathogenesis. Functionally, M42 members have been reported to regulate Aβ deposition via multiple distinct mechanisms. Members containing heparan sulfate‐binding domains can interact directly with Aβ and influence its aggregation.^3^, ^5^, ^7^, ^15^ Meanwhile, non‐heparan sulfate‐binding M42 proteins have been implicated in Aβ pathology via alternative mechanisms such as mediating the cleavage and clearance of APP fragments and Aβ peptides,^16^, ^17^ binding to APP, and promoting its non‐amyloidogenic processing,^9^, ^10^, ^11^ contributing to Aβ uptake and intracellular accumulation.^18^ Additional roles have also been reported, including mediation of aggregated and fibrillar tau uptake and degradation,^19^ as well as elevated levels within cerebrovascular amyloid deposits^7^, ^8^ and plaque‐associated microglia,^20^ suggesting a role in blood–brain barrier integrity and microglial activation. Taken together, these reports point to a multifaceted role for M42 in AD pathology, spanning amyloidogenic processing pathways, Aβ aggregation, and modulation of diverse cellular responses.

Given the reported abundance of M42 proteins within amyloid plaques^21^ and the dynamic interaction of microglia with both amyloid and the ECM, we sought to investigate the influence of M42 members on immune responses independent of Aβ. Using a systematic approach, we purified the majority of M42 proteins and examined their effects in hiPSC‐derived tissue‐resident macrophages.^13^ We identified novel roles for full‐length MDK and the ectodomain of TMEFF2 in modulating immune function and intracellular signaling.

MDK is a secreted heparan‐sulfate binding growth factor involved in central nervous system (CNS) development by mediating neuronal adhesion and neurite outgrowth.^22^ Its expression is tightly regulated under hypoxic and inflammatory conditions and is elevated in diverse pathologies, including cancer, autoimmune diseases, and CNS disorders.^22^ In AD, increased levels have been detected in *post mortem* brain samples, where MDK colocalizes with Aβ plaques, as well as in different mouse models and in vitro systems.^7^ Despite these observations, its role in pathology remains largely undefined. In cell‐free systems, MDK directly binds Aβ, influencing its rate of fibrilization.^7^ Additionally, we previously showed that MDK is highly expressed in hiPSC‐derived neurons and astrocytes, but not in microglia, and its knock‐down in neurons reduces Aβ_42_ levels.^23^ Evidence from in vivo studies, however, is conflicting, with MDK overexpression shown to drive AD pathology in CRND8 mice,^7^ while loss in the 5xFAD mouse model has the opposite effects, enhancing Aβ pathology and promoting neuroinflammation.^24^ Here, we have shown that in addition to accumulating in Aβ plaques and influencing their deposition, MDK may also contribute to AD pathology through modulating immune function. We have demonstrated that, unlike its effects on Aβ aggregation, which appear to be shared with its structural homologue, PTN,^7^ MDK selectively impairs phagocytosis. We did, however, observe a shared effect on intracellular Ca^2+^ signaling with both MDK and PTN treatment inducing a strong response. Our results are in agreement with observations in the periphery, where elevated levels of MDK have been linked to attenuated macrophage responsiveness.^25^, ^26^

TMEFF2 is a transmembrane protein that undergoes a two‐stage proteolytic cleavage, during which the ectodomain is shed into the extracellular space. Its role in AD is poorly understood currently. Similarly to MDK, elevated TMEFF2 levels have been linked to AD, and TMEFF2 colocalizes with Aβ plaques in *post mortem* brain tissue.^27^ Additionally, increased TMEFF2 levels were also detected in 5xFAD mice and shown to accumulate not only within amyloid deposits, but also in reactive astrocytes.^9^ In the same study, the authors speculated about a protective effect of upregulated TMEFF2, with full‐length TMEFF2 promoting non‐amyloidogenic APP processing and the ectodomain binding to Aβ oligomers, preventing their aggregation and alleviating associated neurotoxicity in vitro. Moreover, pro‐inflammatory cytokines and enhanced NF‐κB signaling were previously shown to promote TMEFF2 secretion.^28^ Taken together, this suggests a mechanism in which enhanced TMEFF2 shedding from activated astrocytes may reduce Aβ‐induced neurotoxicity and counteract the build‐up of amyloid plaques. In our in vitro model, treatment with the ectodomain of TMEFF2 enhanced phagocytosis and intracellular Ca^2+^ signaling, identifying an additional mechanism in which elevated TMEFF2 levels may be beneficial in AD.

Given previous reports demonstrating that phagocytosis of apoptotic neurons is primarily mediated by TREM2 signaling^14^, ^29^ and involves intracellular Ca^2+^ release downstream of SYK activation,^30^ we also examined the effects of treatment with MDK and the TMEFF2 ectodomain on SYK phosphorylation at baseline and after stimulation. TMEFF2 significantly reduced baseline levels of SYK phosphorylation, while MDK had no effect. The discrepancy between the observed elevated Ca^2+^ levels and the unchanged/reduced levels of SYK phosphorylation suggests that MDK and TMEFF2 modulate immune function via an alternative receptor signaling route independent of TREM2. The lack of effect on SYK phosphorylation we observed following TREM2 stimulation in the presence of both MDK and TMEFF2 ectodomain supported this argument further. Alternatively, the disagreement between the two assays may also reflect the different timescales of these experiments. It is possible that immediate, swift changes in SYK activation evoked by the addition of these proteins were missed during the 1‐h incubation period. Future studies exploring these dynamic effects, as well as the interaction of MDK and TMEFF2 with alternative signaling pathways, could provide mechanistic insights into their role in modulating immune function.

In addition to MDK and the TMEFF2 ectodomain, we also observed a significant effect on phagocytosis following treatment with HTRA1, the DAG1 ectodomain, and GPC5. As for MDK and TMEFF2, these effects were restricted to the uptake of apoptotic neurons. Notably, pretreatment with any of the M42 proteins evaluated did not significantly alter the phagocytosis of zymosan particles. This may indicate a preferential effect on signaling pathways downstream of immune receptors that recognize “eat‐me” signals, such as TREM2^14^ and MER proto‐oncogene, tyrosine kinase (MerTK),^31^ rather than those involved in pathogen recognition, including the lectin‐ and Toll‐like receptors (TLRs), such as the mannose receptor (MFR) and TLR2/6.^32^

Unlike MDK and TMEFF2, treatment with HTRA1, DAG1 ectodomain, and GPC5 had no significant impact on intracellular Ca^2+^ levels. Meanwhile, we observed a significant increase in Ca^2+^ currents following the addition of recombinant full‐length SFRP1, SPON1, PTN, and the ectodomain of SDC4.

In conclusion, this study reports a systematic evaluation of the effects of individual M42 members on cellular processes independent of Aβ. Our findings suggest that M42 proteins secreted or shed into the extracellular space may act as signaling cues that engage with immune cell surface receptors and alter key functions, such as phagocytosis. We highlight a novel role for MDK and the ectodomain of TMEFF2 as regulators of phagocytosis and intracellular signaling. This work, however, remains subject to some limitations. In this study, we employed hiPSC‐derived macrophages generated via a MYB‐independent differentiation protocol.^13^ Although these cells share transcriptional and functional similarities with hiPSC‐derived microglia monocultures,^13^ our interpretations are limited to conserved immune functions and signaling pathways rather than microglia‐specific biology. Future studies employing hiPSC‐derived models of microglia and exploring the effects of M42 proteins across a broader range of signaling pathways and functions, including migration, cytokine release, or even uptake of alternative cargo such as Aβ, may identify additional roles for M42 in modulating microglial responses.

An additional limitation to our study is the use of different expression systems to obtain recombinant M42 proteins. Importantly, our primary conclusions are based on proteins expressed and purified from mammalian cells, retaining physiologically relevant glycosylation patterns. The single exception is HTRA1, which was obtained commercially and expressed in *Escherichia coli*. HTRA1 is a secreted non‐glycosylated serine protease, and the absence of eukaryotic glycosylation is therefore unlikely to confound its observed activity on phagocytosis. It is, however, possible that the lack of activity observed with the Sf9 purified proteins NTN1, Collagen type XI alpha 1 chain (COL11A1), Low‐density lipoprotein receptor‐related protein 1 (LRP1), Quinolinate phosphoribosyltransferase (QPRT; intracellular), 3‐hydroxybutyrate dehydrogenase 2 (BDH2), Renin binding protein (RENBP), and Neurexophilin‐1 (NXPH1) reflects differences in glycosylation or other post‐translational modifications. Further studies using mammalian‐expressed versions of these proteins will be required to fully assess their potential role in modulating immune signaling.

Finally, M42 comprises a co‐expression network of proteins, and our approach did not take into account or assess the potential synergistic effects of the interactions between members. Furthermore, M42 seems to exert non‐cell‐autonomous effects. An example of this is MDK, which we have detected in hiPSC‐derived neurons and astrocytes, but not microglia, despite seeing effects of MDK treatment in our hiPSC‐derived macrophage model. We have developed tools that can enable future studies employing complex cell systems and exploring the effects of the protein network as a whole across multiple cell types and phenotypes. Such studies would provide valuable mechanistic insights into the role of M42 in AD pathology and may potentially lead to the identification of novel therapeutic targets.

## CONFLICTS OF INTEREST STATEMENT

The authors declare that the work in this manuscript was conducted as part of the Emory‐Sage‐SGC‐Jax TREAT‐AD Center, a collaborative research initiative led by Allan Levey. No other conflicts of interest are declared. Author disclosures are available in the Supporting Information.

## CONSENT STATEMENT

This study did not involve human participants or human data. Therefore, consent was not required.

## Supporting information



Supporting Information

Supporting Information

Supporting Information
