# Supplementary material for: Assessing Aβ‐independent effects of Module 42 on immune function in vitro
Source: Alzheimers Dement. 2026 Feb 25;22(2):e71215. doi: 10.1002/alz.71215 (PMC12933249; doi:10.1002/alz.71215)
Supplement: Supplementary file 1 — Supporting Information [file ALZ-22-e71215-s003.docx]

**Supplementary Table 1.** A table summarising Module 42 recombinant proteins expressed and purified by TREAT-AD, and used in this study, including corresponding publish Target Enabling Packages (TEPs).

| **Protein** | **Region expressed** | **Corresponding domain(s)** | **Expression system** | **Target Enabling Package (TEP)** |
| --- | --- | --- | --- | --- |
| **SDC4** | 19-145 aa | ectodomain | Expi293F | <https://zenodo.org/records/7439018> |
| **NTN1** | 39-453 aa | EGF-like domain | Sf9 | <https://zenodo.org/doi/10.5281/zenodo.12120518> |
| **CTHRC1** | 31-243 aa | full length | Expi293F | <https://zenodo.org/doi/10.5281/zenodo.12521332> |
| **GPNMB** | 1-498 aa | ectodomain | Expi293F | <https://doi.org/10.5281/zenodo.7439220> |
| **SLIT2** | 921-1529 aa | EGF-like domain | Expi293F | <https://zenodo.org/doi/10.5281/zenodo.10694457> |
| **SFRP1** | 32-314 aa | full length | Expi293F | <https://doi.org/10.5281/zenodo.8364781> |
| **DAG1** | 29-303 aa | ectodomain | Expi293F | <https://doi.org/10.5281/zenodo.7387999> |
| **FRZB** | 33-325 aa | full length | Expi293F | <https://zenodo.org/doi/10.5281/zenodo.10694553> |
| **COL11A1** | 1570-1806 aa | Fibrillar collagen NC1 | Sf9 | <https://zenodo.org/doi/10.5281/zenodo.12106395> |
| **TMEFF2** | 41-302 aa | ectodomain | Expi293F | <https://zenodo.org/doi/10.5281/zenodo.10694408> |
| **LRP1** | 20-172 aa | Low-complexity domain | Sf9 | <https://zenodo.org/doi/10.5281/zenodo.10694432> |
| **MDK** | 21-143 aa | full length | Expi293F | <https://doi.org/10.5281/zenodo.5903102> |
| **QPRT** | 1-297 aa | full length | Sf9 | <https://doi.org/10.5281/zenodo.7968422> |
| **SLIT1** | 916-1534 aa | EGF-like domain | Expi293F | <https://zenodo.org/doi/10.5281/zenodo.10694504> |
| **BDH2** | 1-245 aa | full length | Sf9 | <https://zenodo.org/doi/10.5281/zenodo.11206148> |
| **RENBP** | 11-427 aa | full length | Sf9 | <https://doi.org/10.5281/zenodo.14594819> |
| **OLFML3** | 123-406 aa | Olfactomedin domain | Sf9 | <https://zenodo.org/doi/10.5281/zenodo.11206136> |
| **NXPH1** | 22-271 aa | full length | Sf9 | <https://doi.org/10.5281/zenodo.14594428> |
| **PTN** | 33-168 aa | full length | Expi293F | <https://doi.org/10.5281/zenodo.7968475> |

**Supplementary Table 2.** A table summarising Module 42 recombinant proteins that failed to express in sufficient quantities and/or quality in-house, and were thus excluded from downstream functional characterisation *in vitro*.

| **Proteins** | **TEPs** |
| --- | --- |
| **SMOC1** | <https://doi:10.5281/zenodo.8364023> |
| **HTRA1** | <https://doi.org/10.5281/zenodo.7968342> |
| **SPOCK1** | <https://doi.org/10.5281/zenodo.14594101> |
| **FLT1** | <https://zenodo.org/doi/10.5281/zenodo.12119055> |
| **ECE1** | <https://zenodo.org/doi/10.5281/zenodo.12124951> |
| **SPON1** | <https://zenodo.org/doi/10.5281/zenodo.11167949> |
| **COL25A1** | - |
| **SPOCK3** | <https://doi.org/10.5281/zenodo.14593794> |
| **SPOCK2** | <https://zenodo.org/doi/10.5281/zenodo.11206030> |
| **NTN3** | <https://doi.org/10.5281/zenodo.14593717> |
| **GPC5** | <https://doi.org/10.5281/zenodo.14593569> |

**Supplementary Table 3.** Endotoxin levels detected in in-house purified Module 42 recombinant proteins.

| **Proteins** | **Endotoxin levels at 1 µg/ml (EU/ml)** |
| --- | --- |
| **SDC4** | 0.0118 |
| **NTN1** | undetermined |
| **CTHRC1** | 0.0158 |
| **GPNMB** | undetermined |
| **SLIT2** | 0.0479 |
| **SFRP1** | 0.0259 |
| **DAG1** | undetermined |
| **FRZB** | undetermined |
| **COL11A1** | undetermined |
| **TMEFF2** | 0.0431 |
| **LRP1** | 0.0186 |
| **MDK** | 0.0135 |
| **QPRT** | 0.0244 |
| **SLIT1** | 0.0428 |
| **BDH2** | 0.0707 |
| **RENBP** | 0.0124 |
| **OLFML3** | 0.0114 |
| **NXPH1** | undetermined |
| **PTN** | 0.1234 |
