# Supplementary material for: Assessing Aβ‐independent effects of Module 42 on immune function in vitro
Source: Alzheimers Dement. 2026 Feb 25;22(2):e71215. doi: 10.1002/alz.71215 (PMC12933249; doi:10.1002/alz.71215)
Supplement: Supplementary file 2 — Supporting Information [file ALZ-22-e71215-s002.docx]

**
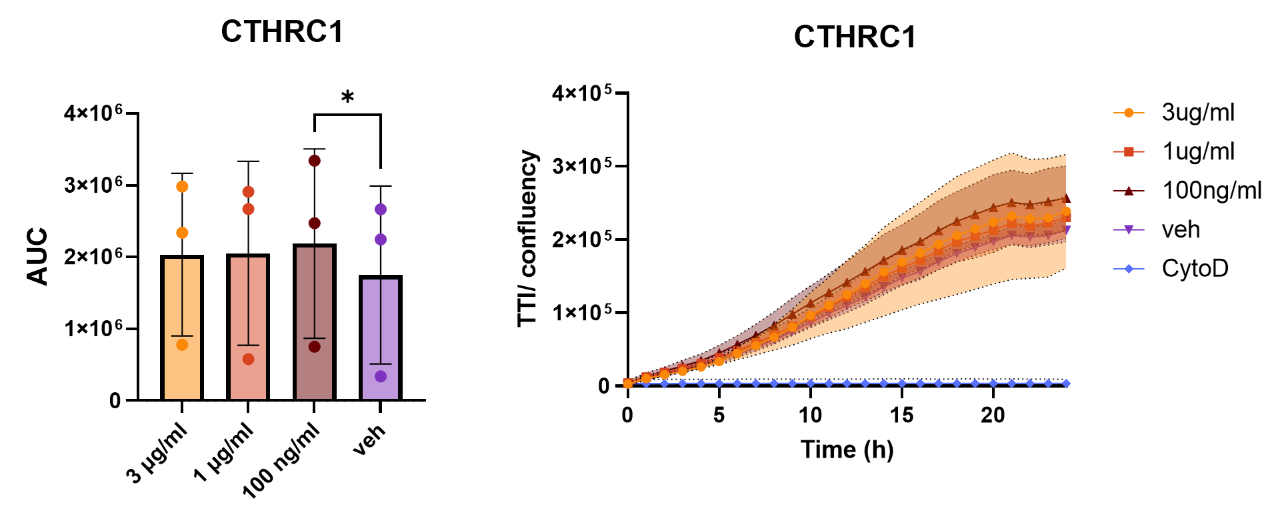
**

**Supplementary Figure 1: Assessing the effect of M42 recombinant proteins on phagocytosis of apoptotic SH-SY5Y cells in hiPSC derived macrophages.** Phagocytosis following treatment with full length CTHRC1. Left panel represents levels of phagocytosis quantified as the area under curve (AUC). Right panel shows a representative trace from one biological repeat (n = 3 technical replicates). Levels of phagocytosis are measured as total integrated intensity of pHrodo-labelled apoptotic neurons normalised to the confluency of macrophage layer (TII/confluency). Cytochalasin D (CytoD, 10 μM) was used as a negative control. N= 3 independent biological experiments (1 independent macrophage factory set-up; 3 technical replicates/ repeat). Error bars correspond to mean ± SD. Repeated measures one-way ANOVA followed by Dunnett’s multiple comparisons test. *P<0.05.


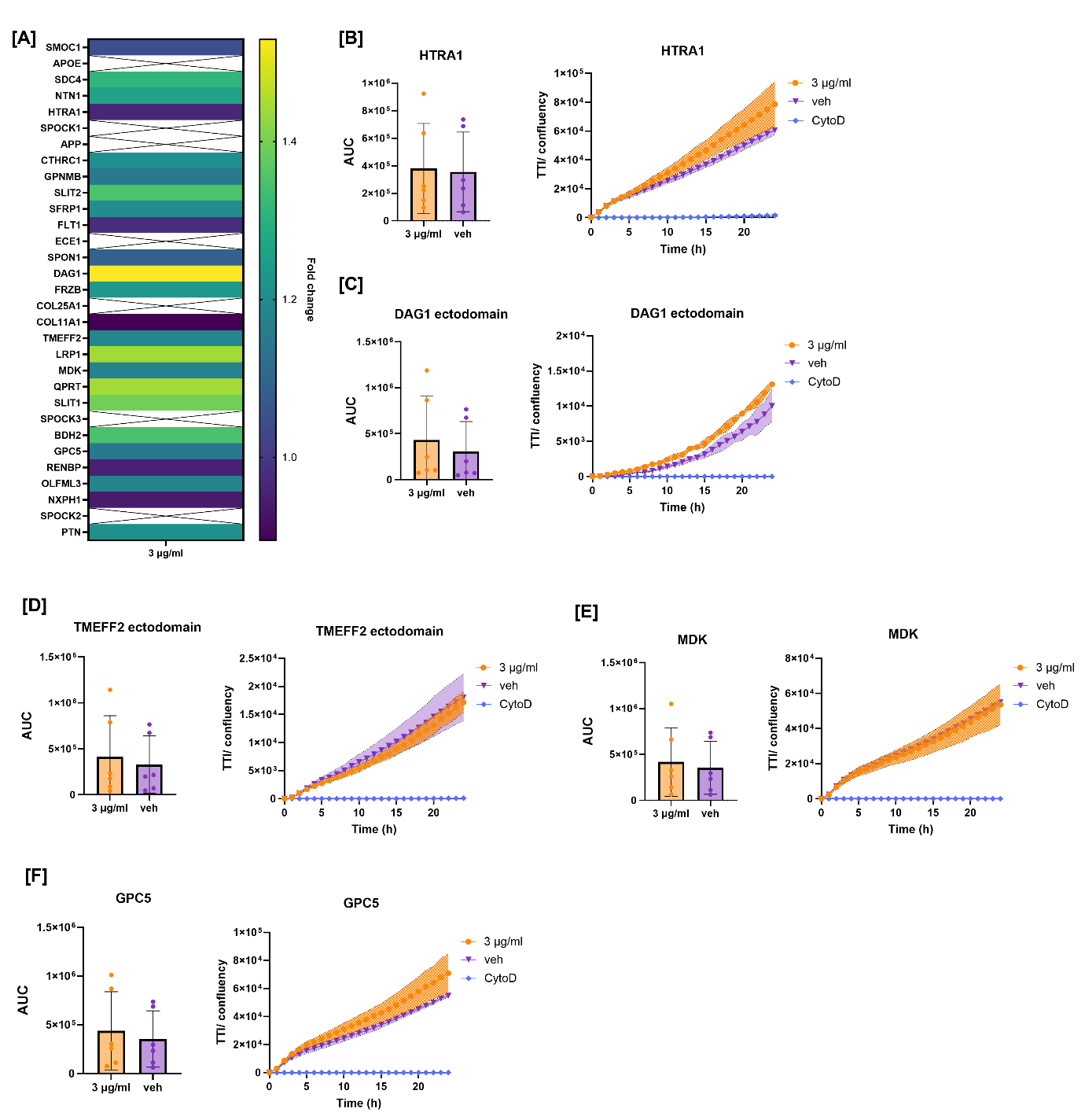


**Supplementary Figure 2: Assessing the effect of M42 recombinant proteins on phagocytosis of zymosan particles in hiPSC derived macrophages.** (A) Summary graph showing the fold change in total phagocytosis assessed over 24 h relative to control (vehicle-treated cells) across all tested proteins at 3 μg/ml. (B-F) Phagocytosis following treatment with HTRA1 (B), DAG1 ectodomain (C), TMEFF2 ectodomain (D), MDK (E), and GPC5 (F). Left panels represent levels of phagocytosis quantified as the area under curve (AUC). Right panels show a representative trace from one biological repeat (n=3 technical replicates). Levels of phagocytosis are measured as total integrated intensity of pHrodo-labelled zymosan particles normalised to the confluency of macrophage layer (TII/confluency). N= 6 independent biological experiments (3 biological repeats from 2 independent macrophage factory set-ups; 3 technical replicates/ repeat). Error bars correspond to mean ± SD. Paired t test.


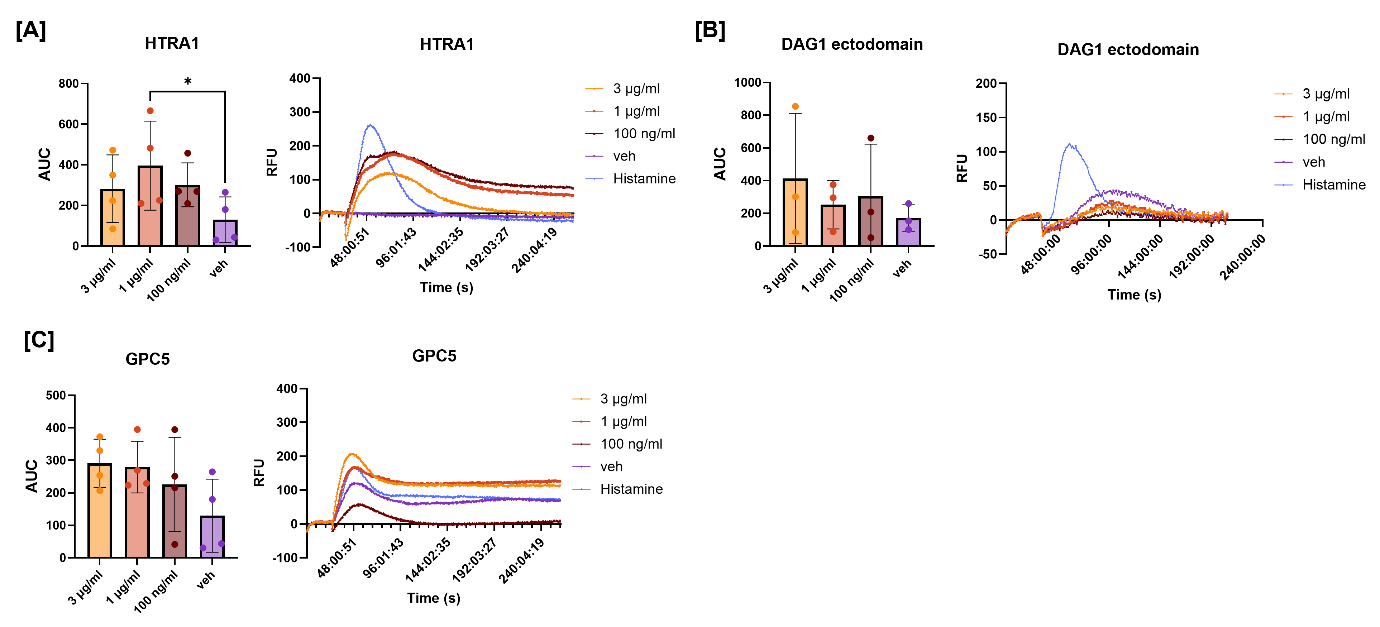


**Supplementary Figure 3: Assessing the effect of M42 recombinant proteins on intracellular Ca^2+^** **signalling in hiPSC derived macrophages.** (A-C) Intracellular Ca^2+^ levels following treatment with HTRA1 (A), DAG1 ectodomain (B), and GPC5 (C). Left panels represent intracellular Ca^2+^ levels defined as the area under curve (AUC). Right panels show representative traces from one biological repeat (n=3 technical replicates). Intracellular Ca^2+^ levels are measured as relative fluorescent units (RFU). Histamine (6 μM) was used as a positive control. N= 4 independent biological experiments (2 independent macrophage factory set-ups; 4 technical replicates/ biological repeat). Error bars correspond to mean ± SD. Repeated Measures One-way ANOVA followed by Dunnett’s multiple comparisons test. *P<0.05.


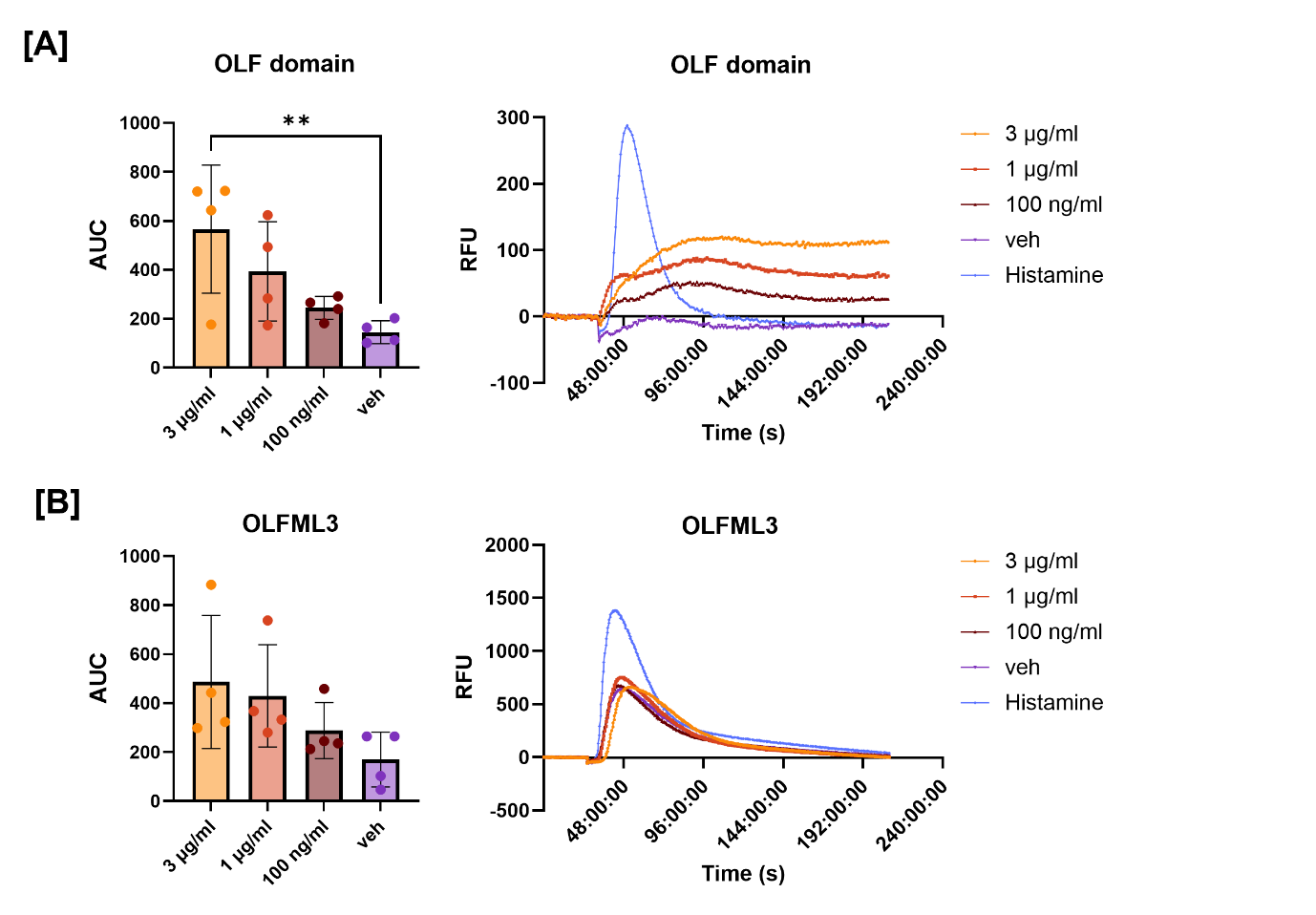


**Supplementary Figure 4: Assessing the effect OLFML3 on intracellular Ca^2+^ signalling in hiPSC derived macrophages.** Intracellular Ca^2+^ levels following treatment with the OLF domain of OLFML3 (A) or full length OLFML3 (B). Left panels represent intracellular Ca^2+^ levels defined as the area under curve (AUC). Right panels show representative traces from one biological repeat (n=3 technical replicates). Intracellular Ca^2+^ levels are measured as relative fluorescent units (RFU). Histamine (6 μM) was used as a positive control. N= 4 independent biological experiments (2 independent macrophage factory set-ups; 4 technical replicates/ biological repeat). Error bars correspond to mean ± SD. Repeated measurements one-way ANOVA followed by Dunnett’s multiple comparisons test.
